# Effectiveness of adding a peak flow meter for the identification of patients with chronic obstructive pulmonary disease in real-world clinical practice

**DOI:** 10.1186/s12890-025-03708-8

**Published:** 2025-05-17

**Authors:** Hideki Ikeda

**Affiliations:** Department of Pulmonary Medicine, Sanyudo Hospital, Fukudamachi 2-1-55, Yonezawa, 992-0033 Yamagata Japan

**Keywords:** COPD, Screening, Peak expiratory flow meter, Real-world clinical practice

## Abstract

Peak expiratory flow (PEF) measurement is useful for detecting moderate and severe chronic obstructive pulmonary disease (COPD). We aimed to validate its combined effectiveness with a questionnaire and to determine appropriate cutoff values in Japan. Outpatients aged ≥ 60 years with a smoking index ≥ 400 cigarette-years receiving non-respiratory treatment and patients with COPD receiving regular treatment underwent PEF measurements and pulmonary function tests. Receiver operating characteristic (ROC) curves were created to differentiate between the percentage forced expiratory volume in 1 s (%FEV_1_) values above or below 80%, based on PEF values normalized by height (method A) and height squared (method B). Of 98 patients, COPD was confirmed in 15 (15.3%). The reference values used to estimate %FEV_1_ < 80% derived from the ROC curve were 2.40 and 1.37 for methods A and B, respectively. Both methods had a high area under the ROC curve (0.92) (*p* < 0.001). The number of suspected COPD (sCOPD) cases was narrowed down from 98 to 33 and 27 using methods A and B, respectively. The combination of the COPD questionnaire and peak flow meter can effectively differentiate severe from the more severe half of moderate COPD from sCOPD cases and improve diagnosis of asymptomatic COPD cases that may require medication prescription by general practitioners.

## Introduction

The prevalence of chronic obstructive pulmonary disease (COPD) in the Japanese population was estimated at 8.6% in a survey conducted in 2000 [1], and the incidence rate was 8.1 per 1000 person-years in men and 3.1 per 1000 person-years in women from 1997 to 2005 [2]. The number of patients diagnosed and treated for COPD was approximately 5% of the total number of patients [1, 2]. Despite the Japanese government’s health policy to increase COPD awareness in 2012 [3], the awareness rate in 2020 was only 28% [4], which was far from the target. Meanwhile, Japanese patients with COPD have become older during this period, and deaths associated with COPD in the < 60-year-old age group have begun to decline [5]. This suggests that patients with COPD can live a long life in Japan if they do not die from comorbidities [5].

For more than 20 years, worldwide educational activities have focused on the early detection of COPD [6]. In this context, the COPD Questionnaire Study Group [7], the International Primary Care Airways Group [8], and the Chronic Obstructive Pulmonary Disease–Population Screener (COPD-PS) [9] questionnaires have been proposed as simple screening tools for COPD. However, as the reference values in these questionnaires are not appropriate for the Japanese population, a questionnaire customized for Japanese patients (COPD-Q) was developed [10]. Similar to the COPD-PS, the COPD-Q is composed of five questions. Screening for COPD with these questionnaires requires confirmation by pulmonary function tests [11]. In addition, in real-world clinical practice, particularly among non-respiratory specialists, pulmonary function tests necessary for COPD diagnosis are not always performed [11, 12], which is believed to be one of the reasons for the lack of advancements in early COPD diagnosis.

Screening for asymptomatic mild to moderate COPD (Global Initiative for Chronic Obstructive Lung Disease [GOLD] grades 1 and 2 [6]) has been reported to contribute minimally to improving quality of life, complications, or mortality [13]. However, the existence of patients with moderate or severe COPD without subjective symptoms has been observed in Japanese surveys [14, 15], and cases of undiagnosed moderate or severe COPD that interfere with the treatment of comorbidities may be encountered. Therefore, detecting patients with moderate and severe COPD who have no subjective symptoms to initiate treatment is urgently needed in real-world clinical practice.

Furthermore, many patients with undiagnosed COPD who regularly visit clinics receive medical treatment from physicians other than respiratory specialists [16]. As such, a pulmonary function measurement method that can be easily performed by non-respiratory specialists is necessary to detect patients with COPD who have no subjective symptoms. In recent years, the use of peak expiratory flow (PEF) has been investigated as a simple COPD detection method in real-world clinical practice [17, 18, 19]. PEF alone has been reported to offer excellent utility in detecting moderate and severe COPD [17, 18] while also being cost-effective [18]. However, to date, no reports have validated the detection of COPD by PEF measurement in Japanese patients.

Therefore, we aimed to investigate the effectiveness of combining PEF measurement with a COPD questionnaire in detecting moderate and severe COPD, especially severe COPD, in Japanese patients and to determine the appropriate cutoff value of PEF for this population.

## Methods

### Patients and procedure

**Fig. 1:**
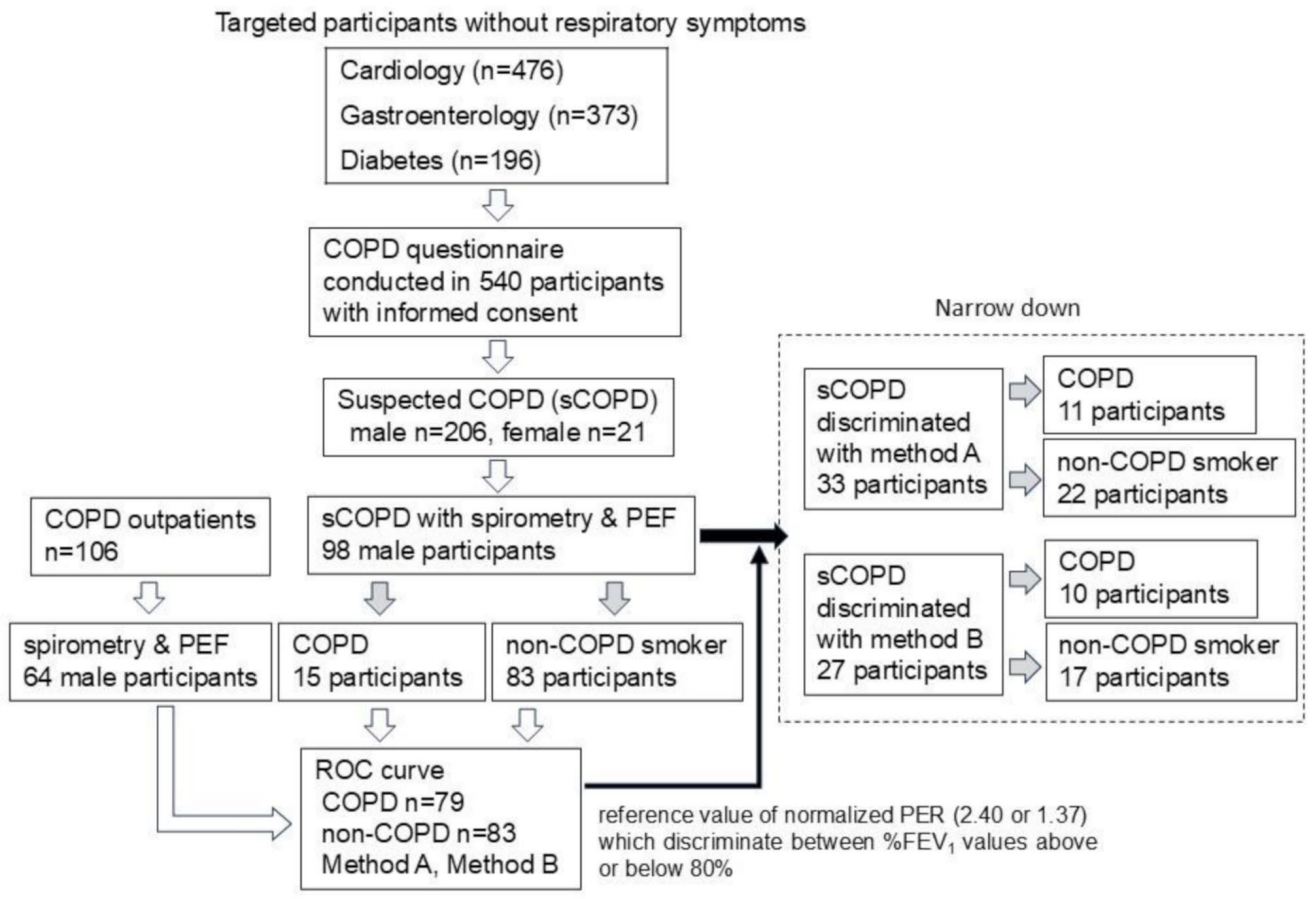
Flowchart for the extraction of unrecognized COPD and smoker-without-COPD (non-COPD smoker) cases from suspected COPD (sCOPD) cases who visited the hospital for non-respiratory treatment, and the sorting of sCOPD cases (within the square with broken lines). Gray arrows indicate COPD and non-COPD identification using the spirometry test. Closed arrows indicate discrimination using the reference value **Abbreviations**: COPD, chronic obstructive pulmonary disease; PEF, peak expiratory flow

From April 2020, all outpatients who visited Sanyudo Hospital were screened for cough, sputum, shortness of breath, and other respiratory symptoms as a precaution against coronavirus disease 2019. Data from these screenings were used to answer the symptom questions in COPD-PS and COPD-Q. Therefore, screening for age and smoking history was added to complete the COPD questionnaire. Figure 1 shows the flowchart for selecting participants.

In both COPD-PS and COPD-Q, a smoking index ≥ 400 (≥ 20 pack-years) equals 2 points, and age ≥ 60 years equals 2 points, for a total score of 4 points, which qualifies for COPD suspicion. This study utilized COPD-Q [10]. As a result of these screenings, the symptom score was zero; only smoking history and age contributed to the score. Thus, all participants aged ≥ 60 years with a smoking index ≥ 400 were assigned to the group suspected of having COPD (sCOPD). Participants in this group underwent spirometry and PEF tests. The spirometry test identified patients with unrecognized COPD, who were then included in the COPD group for receiver operating characteristic (ROC) curve analysis. COPD was diagnosed as a forced expiratory volume in 1 s/forced vital capacity (FEV_1_/FVC x 100 = FEV_1_%) < 70% [4, 5].

In addition, patients with COPD who regularly visited the Department of Respiratory Medicine at Sanyudo Hospital were recruited and assigned to the COPD group. The diagnosis of COPD was confirmed from medical records in accordance with the Japanese Respiratory Society’s COPD Guideline, 6th edition [5]. These patients underwent spirometry and PEF measurement again. Subsequently, the sCOPD and COPD groups were included in the ROC analysis. Using the normalized PEF values, ROC curves were created to differentiate between %FEV_1_ values above or below 80%, which is the cutoff value between mild and moderate COPD [5, 7].

### Sorting of sCOPD with ROC

Among participants in the sCOPD group, those with FEV_1_% ≥70% were allocated to the smoker-without-COPD (non-COPD smoker) group, and the rest were allocated to the unrecognized COPD group. Participants in the sCOPD group were then sorted using the reference value obtained from ROC analysis (Fig. 1).

### Equipment and method

A Mini-Wright^®^ (ATS scale; Clement Clarke International) was used to measure PEF, and a CHESTGRAPH HI-105^®^ (Chest Corporation) was used to test pulmonary function. The predicted FEV_1_ value was obtained using the normal prediction formula of the Japanese Respiratory Society ([FEV_1_ (L)] = 0.036x[height (cm)]-0.028x[age (years)]-1.178) [20]. The percentage of predicted FEV_1_ (%FEV_1_) was obtained for each participant.

Surveys were conducted from May 2023 to October 2023. Patients with a history of lung cancer treatment, pulmonary mycobacteriosis, or asthma were excluded from both the COPD and sCOPD groups.

### Statistical analysis

ROC curves were created, and detectability for the two normalizing methods was compared using Bell Curve for Excel^®^ (Social Survey Research Information Co., Ltd.). Between-group comparisons were made using the Mann–Whitney U test. Changes in the COPD detection rate after adding the peak-flow meter were analyzed using the chi-square test.

### Ethical considerations

This study was approved by the Ethics Committee of Sanyudo Hospital (approval no. 2023-0426-2; April 26, 2023) and performed in accordance with the Declaration of Helsinki and the Ethical Guidelines for Medical and Health Research Involving Human Subjects [21] issued by the Ministry of Health, Labour and Welfare. Written informed consent was obtained from all patients prior to testing.

## Results

The average number of targeted participants who were ≥ 60 years old and not taking any respiratory medication was 1045 per month. The cardiology, gastroenterology, and diabetes departments, along with pulmonology, were the main clinical departments at Sanyudo Hospital. Some patients attended multiple departments, resulting in duplicated counts across departments. Patients ≥ 60 years old constituted 80.1% of the total outpatients in these departments. Figure 1 shows the flowchart for the extraction of unrecognized COPD and smoker-without-COPD.

Additional surveys regarding smoking habits were conducted on 540 patients who provided informed consent (excluding those in the acute or subacute stages of the disease), 227 of whom had a smoking index ≥ 400 cigarette-years and were ≥ 60 years old. Only 21 participants were female; thus, the survey was restricted to male participants (206). Pulmonary function tests were performed on 98 participants (sCOPD group). Of these, 15 participants with obstructive disorders (FEV_1_% [FEV_1_/FVC x 100] < 70%) were transferred to the COPD group, while the remaining 83 were included in the smoker-without-COPD group (gray arrow). These 15 patients with FEV_1_% <70% who frequently visited the hospital were considered to have undetected COPD [22, 23]. Their dyspnea severity was grade 0 according to the modified Medical Research Council classification [24]. Table 1 shows the distribution of airflow obstruction severity in the 15 patients with COPD screened from the sCOPD group.

**Table 1:** Distribution of %FEV_1_ in patients with COPD diagnosed from suspected COPD

	Range for %FEV_1_				
	40–49%	50–59%	60–69%	70–79%	≥80%
Number	1	2	4	6	2

**Abbreviations**: %FEV1, forced expiratory volume in 1 second relative to the reference value; 367 COPD, chronic obstructive pulmonary disease

Meanwhile, the average number of outpatients for pulmonary medicine was 532 per month, and 106 had symptomatic COPD. Sixty-four male patients with COPD underwent pulmonary function tests and PEF measurement. Therefore, 79 participants were allocated to the COPD group, including the 15 patients with COPD from the sCOPD group. The mean age of the smoker-without-COPD and COPD groups was 71.0 ± 8.4 and 75.1 ± 7.2 years, respectively, with the COPD group being significantly older (*p* = 0.001). The smoking index was 793 ± 463 and 1020 ± 528, respectively, and was significantly higher in the COPD group (*p* < 0.001). Patients in the COPD group were classified according to the staging of airflow obstruction [25] (Table 2). Those in the smoker-without-COPD group were also classified by FEV_1_ relative to the reference value (%FEV_1_) (Table 3).

**Table 2:** Distribution of airflow obstruction severity in the COPD group

	Severity of airway obstruction			
	GOLD 4 (very severe)	GOLD 3 (severe)	GOLD 2 (moderate)	GOLD 1 (mild)
Number	11	23	36	9

**Notes**: Severity: GOLD 4, %FEV_1_ < 30%; GOLD 3, 30%≤%FEV_1_ < 50%; GOLD 2, 50%≤%FEV_1_ < 80%; GOLD 1, %FEV_1_ ≥80%

**Abbreviations**: COPD, chronic obstructive pulmonary disease; %FEV1, forced expiratory volume 375 in 1 second relative to the reference value

**Table 3:** Distribution of %FEV_1_ in the smoker-without-COPD group

	50%≤%FEV_1_ < 80%	80%≤%FEV_1_
Number	18	65

Abbreviations: %FEV1, forced expiratory volume in 1 second relative to the reference value; 381 COPD, chronic obstructive pulmonary disease

**Fig. 2:**
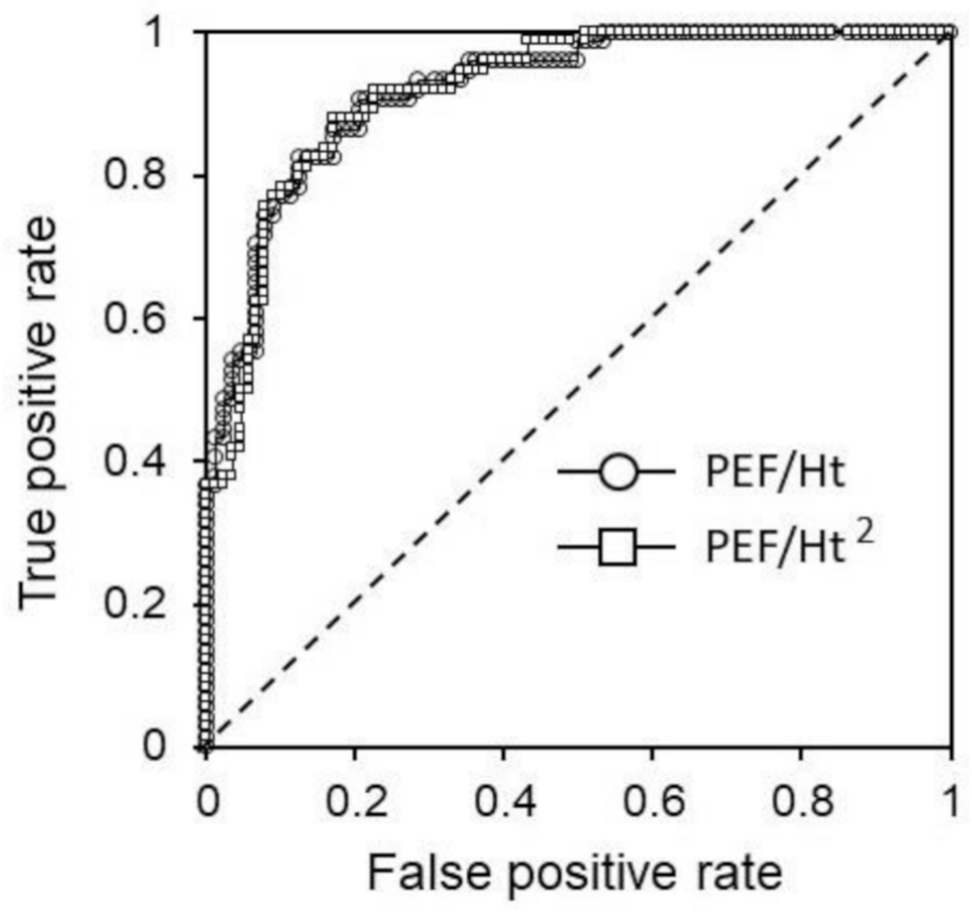
ROC curve analysis for discriminating %FEV_1_ < 80% by PEF/Ht or PEF/Ht^2^ **Abbreviations**: ROC, receiver operating characteristic; %FEV_1_, forced expiratory volume in 1 s relative to reference value; PEF, peak expiratory flow; Ht, height

From the PEF values normalized by height (PEF/Ht) (method A) and height squared (PEF/Ht^2^ × 100) (method B), ROC curves (Fig. 2) were created for each condition to discriminate between %FEV_1_ values above or below 80%, which is the cutoff value between mild and moderate COPD [5, 7]. A previous report [18] used method B for normalization. However, the calculation formula for the standard value of FEV_1_ shows a first-order correlation with height and age in the Japanese population [20]. Thus, method A was employed for normalization in addition to method B. The units for PEF and height were liters per minute and centimeters, respectively. ROC analysis was performed for 161 participants, combining the smoker-without-COPD and COPD groups. ROC analysis showed that both methods (A and B) had an AUC > 0.9, indicating high detectability (*p* < 0.001), with the respective AUCs almost overlapping (Table 4). No significant difference was observed in detectability between the two AUCs (*p* = 0.611; Table 4). The reference values determined from ROC analysis were 2.40 and 1.37 for methods A and B, respectively. The sensitivity and specificity for detecting %FEV_1_ < 80 at these reference values are shown in Table 4.

**Table 4:** Results from the ROC curve analysis for discriminating %FEV_1_ < 80%

	AUC	*p* Value	Reference value	Sensitivity (%)	Specificity (%)
PEF/Ht	0.9203	< 0.001	2.40	84.6	84.5
PEF/Ht^2^ × 100	0.9178	< 0.001	1.37	88.0	81.0

**Abbreviations**: ROC, receiver operating characteristic; %FEV1, forced expiratory volume in 1 387 second relative to the reference value; AUC, area under the curve; PEF, peak expiratory flow; Ht, 388 height

Figure 1 shows the discrimination of sCOPD using the reference values 2.40 and 1.37, which were derived from the ROC analysis of methods A and B, respectively. Table 5 shows changes in the number of targeted participants using methods A or B. The number of participants in the sCOPD group decreased from the initial 98 to 33 (33.7%) and 27 (27.6%) when combined with methods A and B, respectively (gray arrow). Since ROC curves were created for each condition to differentiate between %FEV_1_ values above or below 80%, there was leakage in COPD detection from the sCOPD group: 4 using method A and 5 using method B (Table 5). The lowest %FEV_1_ value among these missed cases was 66.3%. When these reference values were applied to the COPD group (Table 6), 4 patients for method A and 6 patients for method B among the 70 patients with %FEV1 < 80% had a missed diagnosis (false negatives). However, the lowest %FEV_1_ for these missed COPD cases was 67.5%. Therefore, cases of severe and very severe COPD, as well as the more severe half of moderate COPD (50≤%FEV1 < 65), were detected in both the COPD and sCOPD groups without exception.

**Table 5:** Number of suspected COPD cases and true COPD with the spirometry test

	Questionnaire alone	With method A	With method B
Suspected COPD	98	33	27
Non-COPD	83 (84.7%)	22 (66.7%)	17 (63.0%)
COPD	15 (15.3%)	11 (33.3%)	10 (37.0%)
Leaked COPD		4	5

**Abbreviations**: COPD, chronic obstructive pulmonary disease; COPD-Q, COPD questionnaire; 394 FEV, forced expiratory volume; GOLD, Global Initiative for Chronic Obstructive Lung Disease; 395 GP, general practitioner; PEF, peak expiratory flow; ROC, receiver operating characteristic

**Table 6:** Accuracy of COPD classification using methods A and B

	Diagnosed with spirometry	With method A		With method B	
		True	False	True	False
Mild COPD	9	6	3	6	3
Moderate COPD	36	32	4	30	6
Severe COPD	23	23	0	23	0
Very severe COPD	11	11	0	11	0

**Abbreviations**: COPD, chronic obstructive pulmonary disease

## Discussion

The spirometry test revealed that only 15.3% of the 98 participants who were sCOPD based on the questionnaire alone actually had COPD (detection rate = 15.3%). Combining the normalized height (method A) and normalized height squared (method B) criteria with the questionnaire narrowed down the number of sCOPD cases from 98 to 33 (33.6%) and 27 (27.6%), respectively. In addition, after applying the two methods, the number of detected COPD cases among sCOPD cases changed from 15 to 11 and 10, respectively (Table 5). Notably, the lowest %FEV_1_ value for leaked COPD was 66.3%, and the lowest %FEV_1_ for symptomatic COPD with PEF below these reference values was 67.5%. All very severe, severe, and the more severe half of moderate COPD cases (50≤%FEV1 < 65) could be classified within the COPD group (Table 6). Therefore, adding the peak flow meter to the COPD questionnaire can decrease the number of suspected COPD cases without missing very severe, severe, and the more severe half of moderate COPD cases. In clinical practice, screening for asymptomatic mild to moderate COPD contributes minimally to improving quality of life, complications, or mortality [13]. However, some asymptomatic moderate and severe COPD cases require urgent treatment initiation. Thus, adding the peak flow meter to the COPD questionnaire may be an effective case-finding approach for detecting asymptomatic patients with COPD who require immediate treatment in primary care. We consider that all the target cases remaining after sorting out require strong smoking cessation advice and do not need immediate spirometry testing. However, determining the most epidemiologically effective reference value remains a future challenge.

A previous study suggested that a PEF < 80% predicted more than 90% of COPD cases, including moderate and severe cases [26]. Currently, no standard value exists for mechanical PEF in Japanese patients. Therefore, two methods were employed for normalization in this study. ROC curves were then created to discriminate between mild COPD (GOLD grade 1) and more severe COPD (GOLD grades 2, 3, and 4). No differences were observed in detection ability between the two normalization methods. A previous study in a Japanese population reported AUCs of 0.796, 0.747, and 0.775 for COPD-Q, COPD-PS, and IPG, respectively [10]. Another study reported an AUC of 0.82, sensitivity of 90%, and specificity of 50% for the detection of FEV_1_% <70 [26]. Our study showed better results, with an AUC of 0.92, sensitivity of 84.6–88.0, and specificity of 81.0–84.5. The reference value of 1.37 becomes 2.28 when the unit L/min/cm^2^ × 100 is converted to the unit L/s/m^2^, according to a previous study [18]. This value exceeds the reference value of 1.8 used to detect moderate COPD (GOLD grade 2) in another study [18]. This difference highlights the necessity of race adjustment in pulmonary function tests for COPD diagnosis [27]. Further validation and comparison of PEF normalization methods in Japanese patients are needed.

In this study, patients attending the hospital for non-respiratory diseases (mainly cardiology, gastroenterology, and diabetes) were recruited. In Japan, these are the main diseases that commonly present as comorbidities of COPD [23]. The choice of patients in this study is in line with real-world clinical practice [28]. It is also expected that the ratio of undiagnosed COPD cases in general practitioner (GP) outpatient clinics will be as high as that in the present study. Meanwhile, the spirometry test was actively performed by only 27% of GPs in northeastern Japan [29]. The initial costs of new purchases and technical training are significant barriers to the widespread use of spirometry by GPs in Japan and other countries [29, 30]. Moreover, the COPD diagnosis rate in clinical settings is extremely low in Japan [31]. Thus, the Japanese Respiratory Society proposed the COPD questionnaire for screening in GP settings [5]. After COPD screening with the questionnaire, GPs should refer patients suspected of having COPD to a pulmonologist [5]. However, the ability to perform spirometry tests remains limited in hospital pulmonology departments. In such a situation, narrowing down patients suspected of COPD is needed. This study revealed that GPs can effectively identify asymptomatic severe and the more severe half of moderate COPD using a peak flow meter without having to resort to spirometry tests. A GP’s recommendation for patients to stop smoking is the first treatment for mild asymptomatic COPD before medication [5].

This study is the first investigation focusing on the use of peak flow meters for detecting COPD in Japan. Outside Japan, a peak flow meter combined with a questionnaire and mini-spirometer has helped detect asthma and COPD in real-life clinical practice [19].

This study had some limitations. It was conducted at a single facility in a small regional city; therefore, verifying the results in larger populations with variations in climate and lifestyle is essential to obtain appropriate reference values. The prevalence of tobacco consumption among males and females is reported as 25.4% and 7.7%, respectively [32], making data collection from female patients more difficult in Japan. Multi-center studies are necessary to determine appropriate cutoff values for both male and female patients. Considering the availability of several mechanical instruments for PEF measurement, the compatibility of values from different instruments may need to be verified. Awareness of COPD in non-smokers [33, 34] has increased in recent years, and these cases should be evaluated in future studies.

## Conclusion

The reference values for detecting %FEV_1_ < 80% with normalized PEFs divided by height or height squared were 2.40 and 1.37, respectively. The combination of the COPD questionnaire and peak flow meter effectively differentiated severe and the more severe half of moderate COPD from sCOPD and improved clinical efficiency in detecting asymptomatic COPD cases that may require medication prescription by GPs.

## Data Availability

All data generated in this study are available from the corresponding author on reasonable request.
